# Case Report: Giant cell-rich osteosarcoma of the cervical spine in the pediatric age. A rare entity to consider

**DOI:** 10.3389/fsurg.2022.1001149

**Published:** 2022-10-14

**Authors:** Rosa M. Egea-Gámez, María Galán-Olleros, Alfonso González-Menocal, Rafael González-Díaz

**Affiliations:** ^1^ Spine Unit. Orthopaedic Surgery and Traumatology Department, Niño Jesús University Children's Hospital, Madrid, Spain; ^2^ Orthopaedic Surgery and Traumatology Department, Infanta Elena University Hospital, Valdemoro, Spain

**Keywords:** osteosarcoma - pathology, spine, cervical cancer, surgical oncology, reconstructive surgical procedure

## Abstract

**Background:**

Although osteosarcoma is the most common primary malignant bone tumor in children, its location in the axial skeleton is rare, particularly at the cervical spine. Early diagnosis, together with multidisciplinary management, improves survival rates. Safe resection and stable reconstruction are complicated by the particular anatomy of the cervical spine, which raises the risks.

**Case Presentation:**

A 12-year-old male patient presented with cervical pain for several months and a recent weight loss of 3 kg. The complementary workup revealed a large destructive bone lesion in C7 with vertebral body collapse, subluxation, partial involvement of C6 and T1, large associated anteroposterior soft tissue components, and spinal canal narrowing. A biopsy suggested giant cell-rich osteosarcoma (GCRO). After 10 cycles of neoadjuvant chemotherapy, surgical resection was performed through a double approach: anterior, for tumoral mass resection from C6-7 vertebral bodies and reconstruction placing a mesh cage filled with iliac crest allograft plus anterior plate fixation; and posterior, for C7 complete and C6 partial posterior arch resection, thus completing a total piecemeal spondylectomy preserving the dura intact, added to a C5-T3 posterior fusion with screws and transitional rods. Postoperative chemo and radiotherapy were administered. Clinical and radiological follow-up showed disease-free survival and no neurological involvement at 3 years.

**Conclusion:**

An extensive review of the literature did not find any published cases of GCRO of the cervical spine in pediatric patients. This can be explained by the combination of three peculiar conditions: its location at the cervical spine region, the young age, and the GCRO variant.

## Introduction

Primary tumors of the spine are rare, representing between 2% and 8% of skeletal tumors, but should always be considered in the differential diagnosis of back symptoms in children. Within this age group, benign bone lesions such as osteoid osteoma and osteoblastoma prevail (1). Osteosarcoma is the most common primary malignant bone tumor in young patients, frequently arising in the limbs but only rarely in the axial skeleton (3%–5%) (2, 3). In this location, it is more prevalent in the lumbar spine and sacrum and quite infrequent in the cervical spine.

It is common for osteosarcomas of the spine to be initially misinterpreted as benign osteoblastomas since their clinical, radiological, and histopathological characteristics are difficult to differentiate (3, 4), thus, biopsy is essential to conduct a proper approach. Giant cell-rich osteosarcoma (GCRO) is considered an uncommon variant of osteosarcoma (5), representing only about 3% of them (6). This atypical variant is characterized by an abundance of osteoclastic giant cells and a paucity of osteoid tumor (7), leading it to be confused with giant cell tumors (8, 9). Infiltration of adjacent bony trabeculae, focal osteoid deposits, and a Ki67 proliferative index of 20%–30% have been reported to be useful for differentiation from giant cell tumors (8).

This study aims to present a young patient diagnosed with this rare variant of osteosarcoma of the cervical spine, the diagnostic sequence, and multidisciplinary treatment, with a focus on the surgical strategy for oncologic resection and cervical spine reconstruction. In addition, the literature on cervical spine osteosarcoma is reviewed along with a summary workup of the published cases of GCRO.

## Case presentation

A 12-year-old male patient presented with cervical pain with onset several months before and no reported history of trauma or overuse. After a detailed anamnesis, the pain appeared to be both mechanical and inflammatory in nature, causing the interruption of sleep and achieving only slight relief with basic analgesia. The patient also reported a recent weight loss of 3 kg. On examination, his neck appeared tilted to the left side and the pain was localized posteriorly on his lower cervical spine, irradiating to the left arm. Spurling test was negative, with limited motion of the neck and tenderness of the paravertebral muscles bilaterally. Strength and sensitivity were preserved in both upper and lower limbs.

**Figure 1:**
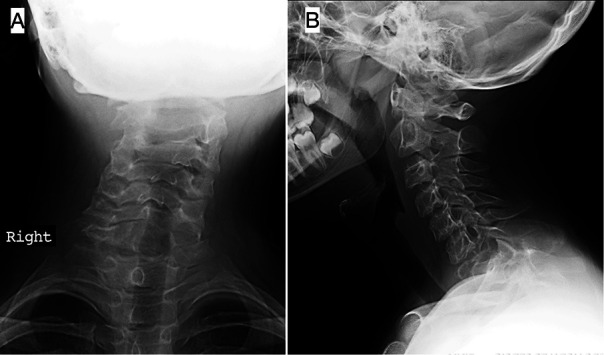
Anteroposterior and lateral radiographs of the cervical spine: (**A**) Osteolytic expansive lesion on C7 vertebral body, with ill-defined borders, expansion and interruption of the upper and lower endplate of the left half of the vertebral body and absent spinous process and left pedicle; also, abnormal cervical alignment in the frontal plane with a slight left-sided tilt of the neck is noted. (**B**) Enlargement of the posterior elements and upper endplate of C7 vertebral body, thinning of the cortical bone, without apparent interruption, periosteal reaction, or soft tissue mass.

**Figure 2:**
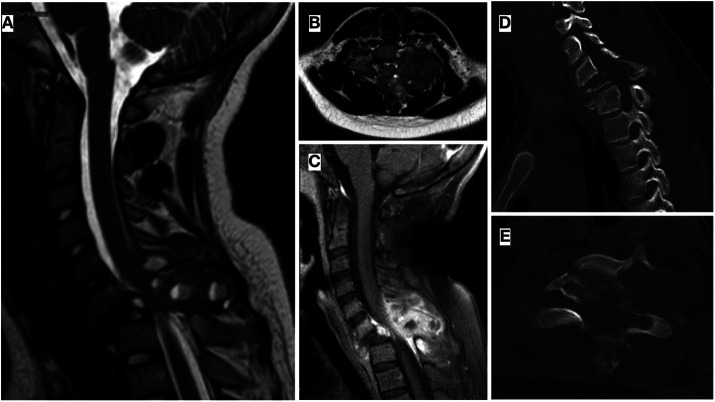
(**A–C**) Sagittal and axial MRI cuts of the cervical spine showing a multiloculated bone lesion in C7 that conditioned vertebral body collapse and subluxation, associated large soft tissue component, with intradural and paravertebral extension, without myelopathy signs. Partial destruction of left posterior elements of C6 and T1 is also visible. (**D,E**) Cervical spine CT revealing a destructive collapsing lesion in C7 vertebra, affecting the posterior part of the vertebral body and the posterior elements, and causing subluxation and angular kyphosis of the cervical spine. There was also partial involvement of the left pedicle, lamina, and spinous process of C6 and, to a lesser extent, the T1 left pedicle.

Simple anteroposterior and lateral radiographs of the cervical spine were taken on the initial visit (Figure 1). Further examination by Computerized Tomography (CT) and Magnetic Resonance Imaging (MRI) revealed a significant destructive bone lesion in C7, with vertebral body collapse, subluxation, partial destruction of the left lamina and spinous process and large associated anterior and posterior soft tissue components. The spinal canal was markedly narrowed at C6–C7, partly due to altered alignment and partly due to invasion by the tumor, obliterating the left posterolateral subarachnoid space (Figure 2). There was also partial involvement of the left pedicle, lamina, and spinous process of C6 and, to a lesser extent, the T1 left pedicle. The left vertebral artery was not visible, suggesting tumoral invasion and blockage which was later confirmed by CT angiography.

A CT-guided percutaneous core needle biopsy was suggestive of GCRO. Extension studies ruled out distant disease, being staged as: stage IIB of the Enneking (10) and American Joint Committee on Cancer (AJCC) systems (11); Tomita type 6, this being an extra-compartmental tumor with adjacent vertebral extension (12) and 1–10/A–D of the Weinstein-Boriani-Biagini (WBB) tumor classification system (13). The Spinal Instability Neoplastic Score (SINS) was 16 points indicating instability.

**Figure 3:**
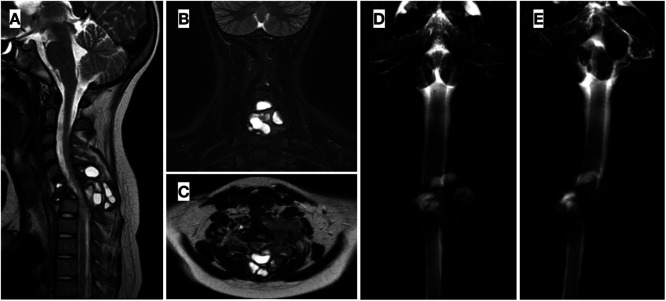
(**A,B,C**) post neoadjuvant chemotherapy MRI showing delimitation and minor shrink of the soft tumor mass. (**D,E**). MR myelography demonstrating significant compromise of the spinal canal, especially at C7 level.

According to the national protocol for localized osteosarcoma in children (SEHOP-SO-2010) (14), the patient underwent ten cycles of neoadjuvant chemotherapy, two of them being omitted due to nephrotoxicity and hepatotoxicity. He remained immobilized during this time with a plain cervical collar. Once neoadjuvant ChT was over, new imaging examinations were done, showing minimal bone tumor size reduction, but delimitation and a slight decrease of the soft tissue component. Also, spinal canal compromise (Figure 3) was evidenced on MR myelography that manifested clinically with increased left radiculopathy.

**Figure 4:**
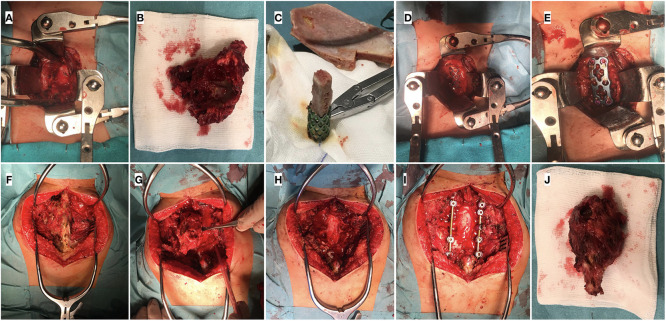
Intraoperative images: (**A–E**). Anterior approach: (**A**) Exposure of anterior part of C6-C7 vertebral bodies; (**B**) Tumoral mass within vertebral bodies; (**C**) Preparation of Moss mesh with iliac crest allograft; (**D**) Placement of mesh with allograft in the cervical spine defect; (**E**) Anterior plate fixation. (**F–J**). Posterior approach: (**F**) Exposure of posterior vertebral elements; (**G**) Posterior vertebral arch resection for total piecemeal spondylectomy; (**H**) Spinal cord and roots decompressed; (**I**) Posterior instrumentation fusion C5-T3 with bilateral screws and rods; (**J**) Posterior part of the tumoral piece.

Consecutively, surgical resection of the tumoral mass and reconstruction (Figure 4) was performed at week 15. Firstly, with the patient in a supine position, a standard left sternocleidomastoid anterior cervical approach was used to expose the anterior longitudinal ligament and C6–C7 vertebral bodies. With a gentle and thorough dissection, laterally to the vertebral bodies, the tumor was found to have invaded the left pedicle and the posterior part of the C7 vertebral body and the inferior part of C6, as seen in the preoperative images. Resection of the tumoral mass in C6 and C7 vertebral bodies was performed followed by an anterior reconstruction, placing a titanium mesh (DePuy Synthes®) filled with iliac crest allograft and completing the first stage fixation with an anterior plate and screws secured to C5 and T1 vertebral bodies (SkylineTM Anterior Cervical Plate, DePuy Synthes®). Subsequently, with the patient in a prone position and through a longitudinal posterior approach, ligaments and muscles were dissected to expose the tumoral mass and C7 posterior elements. A solid cystic tumoral mass was identified in the left paravertebral region, and infiltration of the left posterior arch was also perceived. Wide resection of the posterior arch of C7 and left part of C6 was completed, performing a total spondylectomy of C7 and partial of C6, thereby releasing the spinal cord, preserving the dura intact and verifying bilateral decompression of C6-T1 roots. T1 left pedicle was also resected due to apparent tumoral invasion. To provide spinal stability, adequate alignment, and balance, a C5-T3 fusion was performed, with bilateral screws to the lateral masses of C5 and right C6, as well as pedicle screws to T1 right and bilaterally to T2 and T3 pedicles plus transitional rods (Synapse System, DePuy Synthes®). Drainage was applied and the wound was closed in layers. Neurophysiological monitoring showed no alterations during the procedure, and fluoroscopic control was satisfactory.

**Figure 5:**
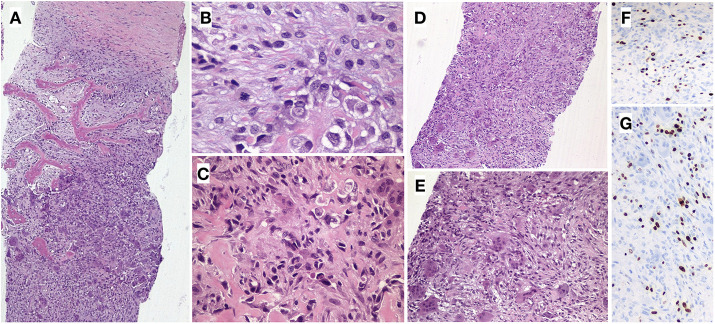
Resected tumor histology of the patient. (**A**) Plexiform and fibrous bone tissue infiltrated by solid neoplastic proliferation (HE, 40x). (**B**) Tumor cells with fusiform and epithelioid morphology with marked cellular atypia and numerous mitotic figures (HE, 400x). (**B,C**) Malignant cells are surrounded by fibrovascular stroma and subtle osteoid deposits (HE, 400x, 200x). (**D,E**) Numerous osteoclastic multinucleated giant cells are identified (HE, 100x, 200x). (**F,G**). Expression of the Ki-67 proliferation marker.

The histopathological results mentioned that the surgical resection piece consisted of a bony tissue measuring 7 × 3.5 × 2.5 cm covered by muscular tissue (Figure 5). Osteoforming neoplastic proliferations infiltrating into adjacent reticular bone trabeculae were identified. They were arranged in a fibrovascular stroma in which numerous osteoclastic multinucleated giant cells could be observed. Ki-67 proliferative index was 26%. A larger piece showed various cystic areas, accompanied by areas of postchemotherapy necrosis that accounted for 30% of the total area of the neoplasm.

**Figure 6:**
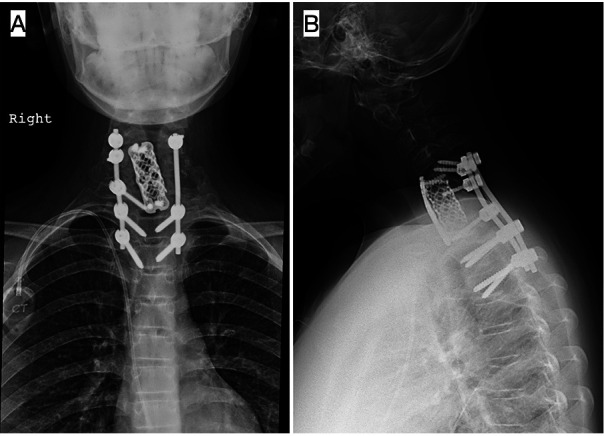
Anteroposterior (**A**) and lateral (**B**) x-rays of the cervical spine at 3 years postoperatively: interbody moss mesh, anterior plate fixation, and bilateral posterior instrumentation fusion with screws and rods from C5 to T3. Adequate alignment and balance, without signs of implant loosening or lack of consolidation.

During the immediate postoperative period, the patient evolved positively, and the surgical wound showed no signs of infection. The patient initiated sitting and ambulation with a rigid cervical collar for two weeks and no neurological symptoms or other complications appeared. Two weeks after surgery, adjuvant chemotherapy was started and subsequently radiotherapy was added as indicated in the proposed protocol. The cervical and upper dorsal spine C4-D1 was irradiated with Volumetric Intensity Modulated Arcotherapy technique with daily Cone Beam CT, 6 MV photons, using isocentric technique, planned with CT at a dose of 50.4 Gy with fractions of 1.8 Gy/fraction. Periodical clinical and radiological visits were set for 1, 3, 6, 12, 24, and 36 months after surgery at the spine clinic, as well as closer visits by the oncologists, with the patient being disease-free to date. In the last evaluation, he was healthy, did not require technical aids for walking or cervical immobilization, and had neither pain nor neurological symptoms. The last radiological examination can be seen in Figure 6.

## Discussion

The case of a rare variant of osteosarcoma located in the cervical spine of a pediatric patient is presented here. A complete initial investigation revealed an aggressive bone lytic lesion located at C7 with soft tissue involvement and compromised spine stability. Distant disease was discarded, and a biopsy confirmed the GCRO diagnosis. Treatment followed the national protocol that combined neoadjuvant ChT, surgical resection and reconstruction, adjuvant ChT, and RT.

After a thorough review of the literature, we were only able to find 42 published cases of GCRO (Table 1), none of which located in the spine, aside from ours. In the current case, infiltration of bone tissue by a solid neoplastic proliferation, subtle osteoid deposits, and a Ki67 proliferative index of 26% help to differentiate it from a giant cell tumor. GCRO is found mostly in children, adolescents, and young adults, with half of the cases analyzed occurring in patients under 20 years old. However, there are reported cases in patients up to 67 years old (23). Most of the reported GRCO occurred in the knee region, 13/42 in the proximal tibia, and 10/42 in the distal femur. Regarding the treatment strategy, 12 out of 42 patients received neoadjuvant chemotherapy, whereas 21 patients were treated with postoperative chemotherapy and only 5 patients received postoperative radiotherapy. Regarding the first surgical treatment, the resection performed was radical in 11 cases, wide in 14 cases, and intralesional in 13 cases. On follow-up, local recurrence was reported in 12 cases and metastasis in 13, with a median follow-up of 36 months.

**Table 1:** Outline of the cases of giant cell-rich osteosarcoma published in the literature.

Study	N	Age	Sex	Location	NA ChT	Resection	Reconstruction	ChT	RT	Local recurrence	Metastases	Follow-up (months)
Barhust 1986 (7)	9	41	F	Femur diaphysis	-	1° Intralesional; 2° Radical	1° Curettage; 2° Disarticulation	-	-	+	+	36
		13	F	Tibia diaphysis	-	1° Intralesional; 2° Radical	1° Curettage; 2° Amputation	-	+	-	-	192
		21	M	Femur diaphysis	-	1°Intralesional; 2° Radical	1° Curettage; 2° Disarticulation	-	+	+	-	108
		12	M	Femur diaphysis	-	Radical	Disarticulation	-		-	+	36
		6	F	Proximal tibia	-	1° Intralesional; 2° Radical	1° Curettage; 2° Amputation	+	-	-	-	84
		16	F	Femur diaphysis	-	1° Wide; 2° Radical	1°Arthroplasty; 2° Disarticulation	+	-	+	+	24
		12	M	Proximal tibia	-	1° Intralesional; 2° Radical	1° Curettage; 2° Amputation	-	-	+	+	24
		20	M	Distal femur	-	1° Intralesional; 2° Radical	1° Curettage; 2° Amputation	+	-	-	-	24
		8	M	Femur diaphysis	-	Radical	Amputation	+		-	-	12
Sciot 1995 (15)	1	26	M	Distal femur	NR	NR	NR	NR	NR	NR	NR	NR
Sato 1996 (16)	1	19	M	Distal femur	+	Wide	Reconstruction + Autograft	+	-	-	-	72
Shuhaibar 1998 (17)	1	32	F	Distal femur	-	1° Wide; 2° Radical	1° Resection; 2° Amputation	+		+	+	NR
Bertoni 2003 (18)	1	19	M	Femur diaphysis	-	Wide	Arthroplasty + Autograft	NR	NR	+	+	240
Shinozaki 2004 (19)	1	17	M	Distal radius	-	Intralesional	1° Curettage; 2° Autograft	NR	+	+	+	41
Hong 2005 (20)	1	29	F	Proximal tibia	+	Wide	Arthroplasty	+	-	-	-	11
Nagata 2006 (21)	1	32	M	Distal femur	-	Intralesional	Curettage + cement	-	-	-	-	20
Kinoshita 2006 (22)	1	16	M	Rib	+	Wide	Soft tissue flap	+	-	-	-	60
Fu 2011 (23)	1	67	F	Mandible	-	Wide	Soft tissue flap	+	+	-	-	12
Verma 2011 (24)	1	56	F	Maxilla	-	Wide	Soft tissue flap	+	+	-	-	NR
Gambaroti 2011 (25)	1	29	M	Distal femur	-	Intralesional	1° Curettage; 2° Resection	+	-	+	-	36
Imran 2012 (26)	1	16	F	Proximal tibia	NR	NR	NR	NR	NR	NR	NR	NR
Kinra 2012 (27)	1	21	M	Femur diaphysis	+	NR	NR	NR	NR	NR	NR	NR
Wang 2013 (6)	9	51	M	Proximal femur	+	Intralesional	Arthroplasty	-	-	+	+	18
		18	M	Proximal tibia	-	Radical	Amputation	+	-	-	-	92
		36	F	Proximal tibia	-	Intralesional	Curettage + cement	-	-	+	-	90
		13	M	Proximal tibia	-	Intralesional	Curettage + cement	-	-	+	+	13
		19	F	Distal femur	-	Radical	Amputation	+	-	-	-	74
		33	F	Proximal tibia	-	Radical	Amputation	+	-	-	-	111
		16	M	Proximal tibia	-	Radical	Amputation	+	-	-	+	20
		15	F	Proximal tibia	-	Radical	Amputation	+		-	-	114
		32	M	Proximal tibia	+	NR	NR	NR	NR	NR	NR	5
Vijayan 2015 (28)	1	19	F	Cuneiform		Intralesional	Curettage + cement filling	+	-	-	-	36
Chow 2016 (29)	8	16	M	Proximal tibia	-	Radical	Amputation	+	-	-	-	110
		26	F	Distal femur	-	Radical	Amputation	+	-	NR	+	14
		12	M	Proximal fibula	-	Radical	Disarticulation	+	-	+	+	21
		33	F	Distal femur	+	Wide	NR	-	-	-	-	48
		15	F	Proximal tibia	+	Wide	NR	-	-	-	-	38
		31	F	Metatarsal	+	Radical	Ray amputation	+	-	-	+	30
		11	M	Metatarsal	+	Wide	NR	-	-	-	-	21
		15	M	Distal femur	+	Wide	NR	-	-	-	-	12
Chobpenthai 2019 (30)	1	11	F	Patella	+	Wide	Rotational flap	-	-	-	-	13
Cahayadi 2019 (31)	1	46	M	Proximal ulna	NR	Wide	Arthroplasty	NR	NR	NR	NR	NR
Current report 2022	1	12	M	Cervical spine	+	Intralesional	Reconstruction + allograft	+	+	-	-	36

F, Female; M, Male; NA ChT, Neoadjuvant Chemotherapy; ChT, Chemotherapy; RT, Radiotherapy; OS, Osteosarcoma; GCRO, Giant Cell-Rich Osteosarcoma; NR, Not registered.

The appearance of osteosarcomas in the cervical region has rarely been reported in the literature. There are only 5 published cases of cervical spine osteosarcoma in pediatric patients and the number would increase by 8 more if our research were to include adult patients (Table 2), for a total of 13 cases. Regarding the treatment strategy, 8 cases received neoadjuvant chemotherapy, while 12 patients were treated with postoperative chemotherapy and 10 patients received postoperative radiotherapy. Even though radiotherapy has not proven remarkably effective at influencing the long-term prognosis of osteosarcomas, the difficulty of achieving free tumoral resection margins at this anatomical location justifies the necessity of adding radiotherapy as a supplemental therapeutic tool. The histological type of osteosarcoma was telangiectasic in 3 cases, osteoblastic in 2 cases, and chondroblastic, giant cell osteosarcoma, and osteoblastoma type in one case each, with 6 other cases in which the type of osteosarcoma was not registered. As previously mentioned, to date there have been no published cases of GCRO located at the cervical spine. At a median follow-up of 44 months in the published cases, there were 4 local recurrences and 4 metastases.

**Table 2:** Summary of the cases of cervical spine osteosarcoma reported in the literature.

Study	N	Age (y)	Sex	Cervical spine location	NA ChT	Resection	Approach	Reconstruction	Pathology	ChT	RT	Local recurrence	Metastases	Follow-up (months)
Gandolfi 1984 (32)	1	39	F	C6	NR	Intralesional	Anterior	Interbody fusion with autograft	Chondroblastic OS	+	+	NR	NR	NR
Ozaki 2001 (33)	1	5	F	C4	+	Intralesional	Anterior	Anterior fusion + bone graft	Telangiectasic OS	+	+	-	-	26
Ponnampalam 2012 (34)	1	62	F	C2-C4	+	Intralesional	NOT	NOT	Osteoblastic OS	NR	+	NR	NR	NR
Turel 2012 (35)	1	15	M	C5	-	Intralesional	Combined	Posterior fusion + auto/allograft	Telangiectasic OS	+	+	NR	-	12
Feng 2013 (36)	6	22	F	C1	+	Intralesional	Combined	Occipitocervical fusion with bone graft + internal fixation	NR	+	-	+	+	44
		41	M	C2-C3	+	Intralesional	Combined		NR	+	-	+	+	75
		58	M	C3	+	Intralesional	Combined	Titanium mesh filled with bone cement ± posterior internal fixation with pedicle screws.	NR	+	+	-	+	39
		50	M	C6	-	Intralesional	Anterior		NR	+	+	+	+	50
		27	M	C7	+	Intralesional	Combined		NR	+	+	-	-	53
		40	F	C7	+	Intralesional	Combined		NR	+	+	-	-	36
Zils 2013 (37)	2	5	F	C4	`-	Intralesional	NR	NR	Telangiectasic OS	+	+	-	-	168
		11	M	C7-T2	-	Intralesional	NR	NR	Osteoblastic OS	+	+	-	-	86,2
Clarke 2016 (38)	1	8	F	C1	+	Marginal	Combined	Occipitocervical fusion+ graft	Giant cell OS	+	-	-	-	16
Current report 2022	1	12	M	C7	+	Intralesional	Combined	Anterior fusion with titanium mesh, bone graft / anterior plate + Posterior fixation	GRCO	+	+	-	-	36

F, Female; M, Male; y, years; OS, Osteosarcoma; NA ChT, Neoadjuvant Chemotherapy; ChT, Chemotherapy; RT, Radiotherapy; GCRO, Giant Cell Rich Osteosarcoma; NOT, Non-operative Treatment; NR, Not registered.

Concerning local tumor management, the definitive treatment for any malignant tumor should be a wide en-bloc resection including surrounding intact tissue, without violating the tumor capsule to avoid the risk of satellite tumor cells being left behind and thus limiting the possibility of recurrence. Total en-bloc resections of tumors at the cervical spine with vertebral artery control or sacrifice of one of them have been described previously (39, 40). However, in some cases, the proximity of the spinal cord and roots and vascular structures can prevent a wide resection, which forces the surgeon to obtain limited margins. Hence, this being a rare location for osteosarcoma, management poses a special challenge. Total en-bloc spondylectomy refers to a resection where the tumor mass together with the vertebral body and posterior elements are removed as a single unit. Although, it is not a synonym for a wide resection since it is usually a marginal type of resection alongside the tumor capsule. On the other hand, a total piecemeal spondylectomy is an intralesional resection where most of the tumor is excised, but some macroscopic tumor cells might be left, usually due to their proximity to noble structures or, as in the case of our patient, due to the need to remove the tumor from two different approaches and in two separate pieces. In all the cervical spine osteosarcoma cases reviewed, an intralesional resection was performed, except for one en-bloc marginal resection (38). Three of them were done by an anterior approach, and seven with a combined anteroposterior approach as in our case.

Following tumor resection, the challenge is to restore the stability, biomechanics, and global alignment of the cervical spine. Most of the cases evaluated were reconstructed with a circumferential fusion. The cases involving the upper cervical spine were handled with an occipitocervical fusion. A special concern in our case was that the tumor was located in the cervicothoracic area, which is a high-stress junctional zone that is exposed to high mobility and does not tolerate bone loss easily. Hence, a stable reconstruction was performed using a combination of anterior and posterior spinal fusion with the addition of a tricortical iliac crest allograft, a titanium Moss mesh, and an anterior plate. Transitional rods and screws were applied in the transition from the cervical to the thoracic spine, and the screw diameter employed was different for the cervical vertebrae (4.5 mm) than the thoracic ones (5.5 mm).

Osteosarcoma of the cervical spine is exceptional (5 cases in children and 8 in adults), as is the GCRO variant in any location (42 cases). To the best of our knowledge, this is the first published case of GCRO in the cervical spine in a pediatric patient, which can be explained by the combination of three peculiar conditions: its location in the cervical spine region, the young age of the patient, and the GCRO variant. Henceforward, despite its unlikeness, this diagnosis should be considered when dealing with a tumor in the spine. In the cervical spine in particular, oncological resection is almost always intralesional due to the proximity of the neuraxis, which is why radiotherapy is often administered. Following oncological resection, the reconstruction phase pursues local stability, regional alignment, and global spine balance, bearing in mind the growing condition of pediatric patients.

## Data Availability

The original contributions presented in the study are included in the article/Supplementary Material, further inquiries can be directed to the corresponding author/s.
